# Macrophage Migration Inhibitory Factor in Protozoan Infections

**DOI:** 10.1155/2012/413052

**Published:** 2012-02-09

**Authors:** Marcelo T. Bozza, Yuri C. Martins, Letícia A. M. Carneiro, Claudia N. Paiva

**Affiliations:** Laboratório de Inflamação e Imunidade, Departamento de Imunologia, Instituto de Microbiologia, Universidade Federal do Rio de Janeiro, 21941-902, Rio de Janeiro, RJ, Brazil

## Abstract

Macrophage migration inhibitory factor (MIF) is a cytokine that plays a central role in immune and inflammatory responses. In the present paper, we discussed the participation of MIF in the immune response to protozoan parasite infections. As a general trend, MIF participates in the control of parasite burden at the expense of promoting tissue damage due to increased inflammation.

## 1. Introduction

The immune/inflammatory response triggered during infection has an essential role in eliminating the infectious agent and in promoting tissue repair [1]. The very existence of multicellular organisms in an environment replete of infectious agents is made possible by an effective immune system, indicating that the ability to control infections has been throughout evolution an important selective pressure to mold the immune system. However, it is not unusual that the tissue damage observed during infectious processes is caused by the immune/inflammatory response itself. Innate immune receptors recognize conserved microbial molecules from all classes of microorganisms [1, 2]. The activation of these receptors elicits selective intracellular signaling cascades that result in the production of cytokines, chemokines, lipid mediators, and reactive oxygen/nitrogen species. Both the intensity and the quality of the inflammatory responses are determined by the detection of combinations of microbial molecules and molecules from host origin such as cytokines, ATP, and ROS [3, 4]. This activation of the immune system is considered essential for pathogen killing but, on the other hand, is also critically involved in tissue damage and sepsis [1–4]. Thus, the pathology of infectious diseases can result either from a direct effect of the infectious agents or from the immune/inflammatory response, both of which can cause metabolic changes, cellular malfunctioning, and cell death. In fact, the pathology of most infectious diseases is the intricate result of these two forces.

Macrophage migration inhibitory factor (MIF) activity was described in the sixties and it is considered one of the first cytokines to be described [5, 6]. The MIF gene was cloned in 1989 using a functional assay based on its ability to inhibit the random migration of macrophages [7]. A major breakthrough in the characterization of MIF was achieved by a remarkable study that identified proteins secreted by the pituitary gland upon stimulation by LPS [8]. Among these proteins was MIF, and the authors went on to show that blockade of MIF protected mice from LPS-induced lethality, indicating its prominent proinflammatory role in endotoxemia. These studies led to renewed scientific interest on the biology of MIF and opened research avenues in several fields. In the 20 years of research following the cloning of MIF a complex scenario of its biology has emerged and it is now clear that MIF is an important inflammatory mediator that participates in both innate and adaptive immune responses [9].

**Figure 1:**
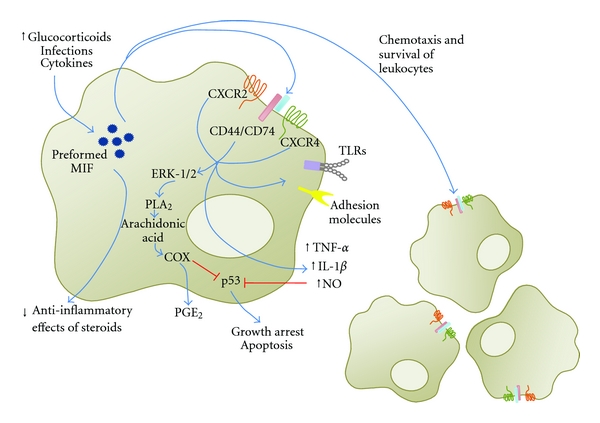
The effects of MIF on macrophage activation. Release of preformed MIF induced by different types of stimuli, such as infections, cytokines, and variations on glucocorticoid levels, has paracrine and exocrine effects: triggering of the CD44/CD74 receptor complex and the CXCR2 and CXCR4 chemokine receptors results in the production of tumor-necrosis-factor-*α* (TNF-*α*), interleukin-1 (IL-1), and nitric oxide (NO,) as well as of arachidonic acid and eicosanoids through the induction of phospholipase A_2_ and cyclooxygenase, and in increased expression of TLRs and adhesion molecules in macrophages. The exocrine effects of MIF include induction of chemotaxis and promoting the survival of leukocytes.

Preformed MIF protein is found in many cell types and is released in response to different stimuli, such as infections and cytokine stimulation [9]. Physiological increases in glucocorticoid concentrations induce immune cells to secrete MIF, and, once released, MIF can counterregulate the anti-inflammatory effects of steroids on cytokine production [10, 11]. The pro-inflammatory activities of MIF include the induction/production of inflammatory mediators such as tumor necrosis factor (TNF), interleukin-1 (IL-1), and nitric oxide (NO) by macrophages, the production of arachidonic acid and eicosanoids through the induction of phospholipase A_2_ and cyclooxygenase via a protein kinase A and ERK-dependent pathway, the increased expression of TLRs and adhesion molecules, antagonistic effects on glucocorticoids activity, and its role as a chemoattractant and in promoting the survival of leukocytes (Figure 1) [12–19]. These effects of MIF are, at least in part, mediated by activation of the CD74-CD44 receptor complex, as well as of the CXCR2 and CXCR4 chemokine receptors (Figure 1) [18–21]. MIF also increases macrophage survival through inhibition of p53 activity, thus reducing activation-induced apoptosis [22]. Interestingly, the inhibitory effect of MIF on p53 is dependent on COX-2 and autocrine production of PGE_2_ by macrophages [17]. This increased survival of macrophages promoted by MIF might affect the immune response to intracellular parasites.

Studies using antibody neutralization, antagonists, or gene deletion demonstrated that MIF plays a critical role in the pathogenesis of several inflammatory disorders, such as sepsis, glomerulonephritis, arthritis, colitis, encephalomyelitis, atherosclerosis, and asthma [8, 9, 14, 18, 23–27]. Indeed, MIF has been shown to influence the pathogenesis of infectious diseases, participating in the protective immune response or playing a critical role in its immunopathogenesis [8, 9, 14, 19, 28–35]. Similarly, polymorphisms of the human MIF gene have been associated with increased susceptibility to or severity of a number of inflammatory diseases [36]. In the present paper we discuss the role of MIF in the host-parasite interaction upon infection caused by protozoan parasites (Table 1).

## 2. The Role of Host MIF in the Pathogenesis of Malaria

Malaria is caused by protozoan parasites from the genus *Plasmodium*. Presently, it is accepted that five species can cause disease in humans: *Plasmodium malariae*, *Plasmodium vivax*, *Plasmodium falciparum*, *Plasmodium ovale*, and *Plasmodium knowlesi* [48]. Together, these species are responsible for around 225 million cases of malaria and nearly one million deaths per year [49]. Although all five species can cause severe diseases [50], *P. falciparum* infections are the most prevalent in the world and are the most likely to complicate, which makes this species responsible for over 90% of the deaths [49]. Severe malarial anemia (SMA) and cerebral malaria (CM) are the most common and life-threatening complications caused by *P. falciparum* infections [51].

## 3. Host MIF Is Detrimental in Experimental Malaria

Experimental murine models of malaria infection have provided an invaluable resource for studying the role of inflammatory and immune responses in the pathology of malaria [52]. Infections caused by the four rodent parasite species (*P. chabaudi*, *P. yoelii*, *P. berghei*, and *P. vinckei*) vary in virulence and pathology depending on the strains of *Plasmodium *and laboratory mouse used [52]. For example, BALB/c mice develop a lethal infection with rapidly increasing parasitemia and anemia that peak approximately on day 8 of infection when inoculated with *P. chabaudi chabaudi* AS [53]. For this reason, this parasite-mouse combination is considered an experimental model of SMA. On the other hand, the same strain of mouse develops a nonlethal self-resolving infection with peak parasitemia also on day 8 of infection followed by cell-mediated parasite killing and total parasite clearance on day 15 when inoculated with *P. chabaudi adami* DK [43]. This second model is considered suitable to study the interactions between macrophages and T cells involved in parasite elimination. Alternatively, C57Bl/6 mice develop a neurologic syndrome similar to human CM and characterized by ataxia, convulsions, and coma upon infection with *P. berghei* ANKA or *P. yoelii* 17XL [54–56]. Interestingly, *P. yoelii* 17XL, but not *P. berghei* ANKA, also induces CM when inoculated in BALB/c mice [52].

However, none of the rodent *Plasmodium* strains are natural pathogens of laboratory mouse strains and the course of infection and complications observed in some mouse-parasite combinations including SMA and CM differ from the human spectrum of disease [52]. For instance, peak anemia in the *P. c. chabaudi* AS-BALB/c model correlates with a peak parasitemia of around 20%, which makes the destruction of infected erythrocytes a major contributor to the physiopathogenesis of anemia in this model [52, 57]. Although it also occurs in acute hyperparasitemic infections, the development of SMA in humans occurs mainly in chronic infections with low parasitemias (<5%) and appears to be more related to other mechanisms such as the destruction of uninfected erythrocytes and the suppression of the erythropoietic response [57]. The majority of mouse models of CM are characterized by the adhesion of leukocytes, instead of infected erythrocytes in the brain microvasculature as occurs in human CM [58].

Studies using mouse models of malaria indicate that MIF plays a detrimental role during the infection [43, 44, 53]. *Mif^−^/^−^* mice in the BALB/c background or animals treated with anti-MIF neutralizing monoclonal antibodies are more resistant to *Plasmodium chabaudi adami* infection than wild-type controls presenting a significant reduction in peak and cumulative parasitemia [44]. Accordingly, the infection of BALB/c mice with *P. chabaudi chabaudi* AS, a mouse model of SMA, revealed that elevated concentrations of MIF in the plasma are associated with severity of anemia and suppression of erythropoiesis [43, 53]. In addition, *Mif^−^/^−^* mice infected with *P. c. chabaudi* develop a parasitemia curve similar to that of wild-type controls but present less severe anemia, less inhibition of erythroid colony formation, and a higher survival [43].

It is not clear why blockade of MIF reduces parasitemia during *P. c. adami* but not *P. c. chabaudi* infection. As the development of immunity and/or anemia in mouse and human malaria result from a complex process that involves multiple factors [57, 59], these findings indicate that MIF could act modulating different mechanisms during *Plasmodium* infection. For example, MIF attenuates the development of Th1 responses following *P. c. adami* infection in BALB/c mice by decreasing T CD4^+^ IFN-*γ* production and enhancing IL-4 [44]. Nevertheless, experimental evidence has suggested no role for IFN-*γ* and TNF as inhibitors of erythropoiesis in mice [60] and serum concentrations of these cytokines are the same during the critical period of anemia in *Mif^−^/^−^* and wild-type mice infected by *P. c. chabaudi* [43], indicating that the role of MIF in this mouse model of SMA is independent of the contribution of TNF or IFN-*γ*.

Production of MIF in mouse SMA seems to be triggered by hemozoin, which is an insoluble heme polymer produced by parasite catabolism of host hemoglobin [43, 53]. Hemozoin contributes to the suppression of erythropoiesis in several ways, including the induction of MIF [61–63]. Mouse macrophages secret MIF in response to *P. c. chabaudi*-infected erythrocytes and synthetic hemozoin in a dose response manner [43, 53]. MIF inhibits erythroid colony formation and differentiation in mouse and human bone marrow cell cultures containing erythropoietin by modulating MAP kinase activation [10, 43]. Taken together these data indicate that MIF plays a role in the physiopathology of mouse SMA by decreasing red blood cell production during the infection.

Nevertheless, the role of MIF in semi-immune mouse models of SMA (multiple cycles of *P. berghei* ANKA infection followed by antimalarial treatment in C57Bl/6 mice) that present low levels of parasitemia during anemic episodes and are believed to be more closely related to the human pathology [64, 65] was not investigated yet. It is also still unclear what is the relationship, if any, between MIF production during *Plasmodium* infection in mice and other mechanisms known to be involved in mouse SMA such as lysis of infected erythrocytes due to schizogony and destruction of noninfected erythrocytes [66], by phagocytosis [64], and auto-antibodies [65].

## 4. Host MIF Seems to Be Protective in Human Malaria

Studies conducted in Africa have reported lower concentrations of MIF in *P. falciparum*-infected children when compared to asymptomatic ones [46, 67, 68]. These studies have shown an inverse correlation between MIF concentrations and parasite burden [68] and suggested a protective role for MIF during noncomplicated malaria [67] and human SMA [46]. Furthermore, the data above is corroborated by an experimental work with healthy European volunteers showing that MIF concentrations are decreased in response to early *P. falciparum* infection but are increased in response to antimalarial treatment [45]. Several other reports in children [43, 69] and adults [70, 71] infected with *P. falciparum* showed conflicting results adding to the controversy on the role of MIF on malaria pathogenesis. The reasons behind these discrepancies are not obvious but one should consider that factors such as the previous degree of immunity of the studied population, which can change the pattern of response to the infection [59], and the presence of *Plasmodium*-derived MIF homologues [71] were not accessed and, consequently, not taken into account when analyzing the different studies.

Furthermore, an inverse correlation between MIF plasma concentrations and hemozoin accumulation was showed in children affected by severe anemia indicating that, different from mouse models, hemozoin may decrease MIF production in human malaria [46]. The explanation for this paradox may lie in a feedback loop involving long-lasting hemozoin activation of macrophages in these children. In fact, PBMC from malaria-naïve patients can react to hemozoin by either increasing or decreasing MIF production [46], depending on whether they have a generally well-preserved MIF production. Additionally, MIF polymorphisms also give rise to variable magnitudes of response to hemozoin [43] and could also help to explain the variability found in these studies regarding MIF production in response to hemozoin. Accordingly, there is an association between certain MIF haplotypes of the −173G/C and −794CATT5-8 polymorphisms and susceptibility to SMA [43, 47].

Few studies in humans indicate that MIF is involved in the pathogenesis of cerebral malaria [72–74]. Necropsy studies show a decrease in endothelial cell expression of MIF in brain vessels of cerebral malaria patients when compared to endothelial cells from axillary and chest vessels [72, 73]. A clinical study in India showed that high concentrations of MIF in the plasma are associated with death in cerebral malaria patients [74]. Finally, women with placental malaria infection presented significantly higher levels of MIF in the placental intervillous blood when compared to uninfected pregnant women also indicating a role for this cytokine in malaria infection during pregnancy [69, 75].

## 5. The Role of Plasmodium MIF in the Pathogenesis of Malaria

MIF homologues have been identified in all species of *Plasmodium* examined to date—*P. falciparum *[76–78], *P. vivax* [71], *P. berghei *[54, 79], and *P. yoelii* [55, 56]. The data from the studies cited above indicate that *Plasmodium* MIF (pMIF) is structurally similar to mammalian MIF with around 30% amino acid sequence identity and possesses some, but probably not all, activities normally attributed to the latter. pMIF expression increases with blood-stage parasite maturation: minimal in ring stage and peaking in the trophozoite and schizont stages [54, 55, 76]. There is no evidence showing that pMIF is actively or passively secreted to the blood stream by the parasite, but it is released extracellularly upon schizont rupture, when it becomes available to interact with the host immune cells [54, 55]. Indeed, pMIF has been detected in culture supernatant and plasma of *Plasmodium*-infected mice and humans [55, 56, 67, 71]. However, due to a low conservation of the aminoacid residues in the molecular region known to be involved in the catalytic sites, it seems that the tautomerase and oxidoreductase activities are highly depressed in pMIF when compared to mammalian MIF. Alternatively, pMIF might have a different substrate specificity and the physiological substrate has yet to be identified [54–56].

In terms of functional studies,* in vitro* assays and animal models have shown that pMIF shares some biological properties with mammalian MIF. Indeed, both pMIF and mammalian MIF reduce AP-1 expression, interact with human CD74, induce macrophage chemotaxis, and inhibit erythropoiesis and macrophage apoptosis [54–56, 79, 80]. On the other hand, pMIF does not stimulate the release of IL-8, TNF, or IL-12 from mice and human monocytes or enhance the response of these cells to LPS [55, 78], a key function of mammalian MIF. Finally, a study showed that pMIF and mouse MIF act synergistically to activate the MAPK-ERK1/2 signaling pathway at very low concentrations but act antagonistically at higher concentrations [56], indicating that pMIF and mammalian MIF can interact in a complex way.

The role of pMIF during malaria infection is also not completely understood. Although counterintuitive, studies in mouse models indicate that pMIF attenuates *Plasmodium* virulence by modulating host immune responses [54–56]. C57Bl/6 and BALB/c mice showed a reduction in disease severity when infected with transgenic strains of *P. yoelii* 17X and *P yoelii *17XL that constitutively overexpress *P. yoelii* MIF (PyMIF) [55] or when treated with recombinant PyMIF [56]. This was phenotypically manifested by a decrease in peak and cumulative parasitemia in mice infected with the nonlethal strain *P. yoelii* 17X and prolonged course of infection with a reduction in overall mortality rate in animals infected with the lethal strain *P yoelii* 17XL [55, 56]. On the other hand, the development of cerebral complications in C57BL/6 mice and hyperparasitemia and severe anemia in BALB/c mice did not differ upon infection with *P. berghei *wild-type or *P. berghei *MIF knockout parasites [54]. Once again, studies in humans failed to recapitulate observations from mouse models as pMIF amounts in uncomplicated malaria patients are positively correlated with parasitemia, disease severity, and plasma concentrations of TNF, IL-10, and MCP-1 [71]. Thus, future studies are required to define the role of host and *Plasmodium* MIF in the pathogenesis of malaria.

## 6. Critical Role of MIF in *Toxoplasma gondii *Infection

*Toxoplasma gondii* is an intracellular parasite of the phylum Apicomplexa that is highly adapted to infect different cell types and tissues. *T. gondii* enters its host via the gastrointestinal tract and the innate immune response in the intestine is triggered by the recognition of parasite molecules by enterocytes, macrophages, and dendritic cells (DCs) [81]. The establishment of an antigen-specific Th1 response is essential to protective immunity but also potentially detrimental as excessive intestinal inflammation and tissue necrosis can lead to bacterial translocation and death [81]. The proinflammatory cytokines IL-12, TNF, IFN-*γ*, and IL-1*β* promote resistance against *T. gondii* in part due to the generation of NO by macrophages, an important mechanism responsible for parasite elimination.

A model of systemic infection with *T. gondii *through the intraperitoneal route demonstrated an increased susceptibility of* Mif^−^/^−^* mice when compared to wild-type mice [39]. *Mif^−^/^−^* mice presented higher parasite burden in brains and peritoneal macrophages and reduced plasma concentrations of IL-12, TNF, IFN-*γ*, IL-1*β*, and nitrite during infection [39]. These findings were expected considering that MIF is an enhancer of IL-12 and TNF production by macrophages. A recent study using a model of oral *T. gondii* infection in the BALB/c background also demonstrated an increased lethality and tissue parasitism with reduced IL-12 production and DC activation on *Mif^−^/^−^* mice compared to wild-type mice [41]. DCs obtained from spleens and mesenteric lymph nodes from *Mif^−^/^−^* mice orally infected with *T. gondii* had impaired maturation, with decreased expression of CD80, CD86, CD40, and MHC class II [41]. Thus, the protective role of MIF in *T. gondii* infection is apparently related to the production of proinflammatory cytokines, the activation of DC, and the better control of parasite burden.

BALB/c mice are naturally resistant to oral infection with *T. gondii,* while those of C57BL/6 are highly susceptible displaying intestinal inflammation especially in the ileum [82–84]. This increased lethality of C57BL/6 is related to the extensive intestinal inflammation, tissue necrosis, and a sepsis-like syndrome. Using the peroral route of infection in C57BL/6 mice, it was shown that *Mif^−^/^−^* mice have reduced intestinal and systemic inflammation and survive longer compared to wild-type mice, despite an increase in intestinal parasite burden [40]. Lack of MIF caused a reduction of TNF, IL-12, IFN-*γ*, and IL-23 and an increased expression of IL-22 in ileal mucosa. Signs of systemic inflammation including the increased concentrations of inflammatory cytokines in the plasma and liver damage were less pronounced in *Mif^−^/^−^* mice compared to wild-type mice [40]. Although MIF has been regarded as essential in host protection during *T. gondii* infection, these findings demonstrated a pathogenic role of MIF in natural *T. gondii* infection in susceptible hosts. This dichotomy seems to depend on the route of infection and the genetic background of the host. Thus, MIF is necessary to control parasite burden in resistant and susceptible hosts, but it increases intestinal tissue damage causing death in susceptible hosts while it is essential for survival in resistant hosts.

A major consequence of human *T. gondii*-infection is the severe congenital malformations when the primary infection occurs in the first trimester of pregnancy. A series of studies demonstrated a putative role of MIF on placental biology upon infection with *T. gondii*. Infection or stimulation of chorionic explants with molecules of *T. gondii*, IFN-*γ*, and IL-12 evoked the secretion of MIF [85, 86]. MIF and its receptor, CD74, are present in the syncytiotrophoblast layer and mesenchyme [86]. MIF induces ICAM-1 expression increasing the interaction of villous explants with monocytes [85]. These results suggest that MIF, by influencing the recruitment of *T. gondii *infected monocytes, could facilitate the dissemination of the infection into the deep placental tissues or increase the tissue damage due to inflammation. The same group recently demonstrated, however, that MIF is important for control of placental *T. gondii* infection in first trimester of pregnancy [86].

## 7. MIF Is Protective in *Leishmania* Infection

Leishmaniasis, caused by the protozoan parasites from the genus *Leishmania*, comprises a large spectrum of clinical manifestations including benign ulcer, destructive muco-cutaneous lesions, disseminated cutaneous lesions, and systemic visceral forms [87]. In the mammalian host, *Leishmania sp*. is an obligatory intracellular parasite infecting mainly macrophages. Parasite killing requires macrophage activation with ensuing NO and ROS production [87]. Infection with *Leishmania major* causes skin lesions, which in general parallel the parasite load. A highly polarized Th1 response is effective against *L. major*, activating macrophages to produce NO and resulting in resolving skin lesions. Addition of MIF to macrophage cell cultures results in increased *L. major* elimination [37]. Though the MIF concentration required to reduce *L. major* burden in macrophages is high (1 *μ*g/mL, 100 times that of other cytokines with leishmanicidal effects, such as IFN-*γ*), this concentration is within the range reached in inflammatory conditions. The MIF-induced leishmanicidal effect requires the production of TNF and NO by infected macrophages, and can be reversed by the addition of IL-10, TGF-*β* or IL-13, indicating that it depends on an M1 activation status [37]. The expression of MIF increases during *L. major* footpad inoculation in popliteal lymph node, but the kinetics of its expression compared to that of MIF secretion by T cells upon antigenpresentation suggests that lymph node MIF comes from another cellular source [37]. Consistent with the observed role of MIF as an enhancer of macrophage leishmanicidal function, oral administration of *Salmonella typhimurium* transfected with MIF reduces the size of skin lesions [88], while *Mif^−^/^−^* mice are highly susceptible to *L. major*, developing severe skin lesions late after infection [38]. MIF does not affect Th polarization in *L. major* infection, as indicated by the similar IFN-*γ* and IL-4 production among T cells from *Mif^−^/^−^* and wild-type mice. However, IFN-*γ*-activated macrophages from *Mif^−^/^−^* mice infected *in vitro* with *L. major* have slightly decreased parasite clearance [38], indicating that either they are somewhat insensitive to IFN-*γ* or MIF production is partially required as an intermediary step to IFN-*γ*-induced leishmanicidal activity. The contribution of MIF produced by CD4^+^ lymphocytes to protective immunity against cutaneous leishmaniasis was demonstrated using a model of vaccination with the *L. pifanoi* antigen P-4 [89]. BALB-c mice immunized with P-4 expressed around 10-fold higher amounts of MIF, TNF, and IFN-*γ* mRNAs than the adjuvant controls. Moreover, blockage of MIF with anti-MIF antibody significantly reduced the leishmanicidal ability of macrophages cultured with CD4^+^ lymphocytes obtained from P-4-immunized mice.

 Patients with visceral leishmaniasis due to infection with *L. donovani* presented CD4^+^ lymphocytes expressing low amounts of CD2, IFN-*γ*, and MIF [90]. Antileishmanial treatment caused immunological recovery with increased expression of CD2 and production of MIF. On the other hand, a recent study demonstrated that patients with visceral leishmaniasis caused by *L. chagasi* have increased plasma concentrations of MIF [91]. The MIF concentrations were higher in patients with the active form compared to patients in remission. Interestingly, the authors identify an increase of LPS in the plasma of patients with active disease and the LPS concentrations positively correlated with MIF.

## 8. Identification of Leishmanial MIF and Its Role in Infection

The complete genome sequencing of *L. major *revealed two genes with significant sequence similarities to human MIF (22% identity) [92]. Cloning and expression of one of these leishmanial orthologues of MIF allowed detailed functional and structural characterizations [93, 94]. The X-ray crystal structure of Lm1740MIF/LmjMIF1 demonstrated an overall global topology similar to that of human MIF, but the catalytic site has substantial differences that correlate with the low tautomerase activity of Lm1740MIF/LmjMIF1 and the lack of inhibitory effect of ISO-1, a MIF antagonist that binds to the catalytic site [93, 94]. Similar to the other MIF structures, the *L. major* orthologue proteins adopt trimeric ring architecture. Lm1740MIF binds to CD74, the MIF receptor, indicating a putative role of *L. major* MIF affecting host immunity [93]. In fact, LM1740 induces a signaling cascade on monocytes dependent on CD74 and similar to the one triggered by mammalian MIF. This includes the ability of *L. major* MIF orthologues to induce ERK1/2 phosphorylation, to cause the reduction of Ser^15^-p53 in the cytoplasm, and to protect macrophages from NO-induced apoptosis [93]. Since the macrophage is the main cell type hosting *Leishmania*, the ability of *L. major* MIF to increase the survival of macrophages might represent an important selective advantage that guarantees more efficient amastigote replication.

## 9. MIF Is Protective in *Trypanosoma cruzi *Infection

*Trypanosoma cruzi* is an intracellular protozoan that can infect many cell types, including macrophages. The effective response to *T. cruzi* comprises innate activation of macrophages to induce NO production and, ultimately, the establishment of antigen-specific Th1 CD4 and CTL CD8 responses [95]. Mice genetically deficient in *Mif *also are more susceptible to *Trypanosoma cruzi* infection [42]. This increase in susceptibility is accompanied by decreased plasma concentrations of IL-12 and IFN-*γ* along with acute infection and also decreased IL-12 and IFN-*γ* production by splenocytes stimulated with *T. cruzi* antigens early in the acute phase, indicating that in contrast to the trypanosomatid, *L. major*, MIF participates in Th1 polarization in *T. cruzi* infection. This deficient Th1 polarization is reflected by decreased titers of anti-*T. cruzi* IgG2a (but not IgG1). Also, *Mif^−^/^−^* mice have decreased plasma concentrations of TNF, IL-1*β*, and IL-18, suggesting that decreased production of proinflammatory cytokines underlies their susceptibility to *T. cruzi* infection. The deficient Th1 polarization, specific IgG and pro-inflammatory cytokine secretion are all highly compatible with susceptibility to *T. cruzi* infection, but there is currently no functional data to support this hypothesis. In fact, IFN-*γ*-activated macrophages have a prominent role in *T. cruzi* clearance through NO production, a function that can be enhanced by TNF production. As MIF controls TNF production by macrophages in a number of cases and, along with TNF, enhances production of NO by macrophages and the elimination of trypanosomatid *L. major *[37], it seems likely that MIF enhances macrophage trypanocidal activity. Interestingly, increased expression of MIF was observed in myocardium and skeletal muscles from acutely *T. cruzi* infected BALB/c mice and positively correlated with parasite burden and myopathic alterations [96].

A prior intracellular infection can sensitize the organism to septic shock by priming monocytes to overreact in the presence of very low amounts of TLR ligands, as happens in influenza [97], VSV [98], LCMV infection [99], among others. *T. cruzi*-infected mice are highly susceptible to systemic inflammation, which can be caused by infection itself in mice lineages that develop severe inflammatory response or by administration of TNF, anti-CD3 [100], SEB [98], or LPS [101]. The lethal synergism between *T. cruzi* infection and LPS inoculation likely results from redundant lethal pathways induced by TNF and MIF: although both *Mif^−^/^−^* and *Tnfr1^−^/^−^* infected mice succumb to LPS administration, treatment with anti-MIF rescues *Tnfr1*-deficient mice from lethal shock [102]. However, at present there are no studies demonstrating a contribution of MIF to human mortality in Chagas disease.

Almost no information is available on MIF biology in Chagasic patients. The only study that addressed this issue demonstrated that the MIF-173G/C polymorphism confers susceptibility to Chagas disease in two cohorts from Colombia and Peru [103]. Future studies are essential to characterize the participation of MIF in the physiopathology and immunity to *T. cruzi* infection.

## 10. Future Directions

In this paper we described the involvement of MIF in several models of protozoan infections, considering common themes and certain peculiarities specific to each parasite. In general, MIF seems to participate in the control of parasite burden but, in many cases, with the cost of promoting tissue damage due to increased inflammation. The essential role of MIF in the pathogenesis of infectious diseases and, consequently, the concept that it might be used as therapeutic target still require extensive clinical studies. Thus, for the years to come, several aspects of the biology of MIF and its participation in the response to infectious diseases, including parasitic diseases, need to be addressed opening up new highways of research and, possibly, novel therapeutic strategies.

## Figures and Tables

**Table 1:** Role of MIF in the control of parasite burden and in the pathogenesis of protozoan infections.

Intracellular pathogen	Experimental system of MIF manipulation	Effects of MIF on parasite burden	Control of parasite burden	Pathogenesis	Ref.
*Leishmania major*	Murine macrophages, anti-MIF, rMIF	↓	MIF increases macrophage activation through enhancement of TNF and NO production	—	[37]
	*Mif^−^/^−^* mice (C57BL/6)		MIF decreases lesion sizes and mediates leishmanicidal effects of IFN-*γ* on macrophages, reduces their NO and ROS production, but does not alter Th1 polarization	MIF decreases lesion sizes, a finding associated with decreased parasite burden	[38]

*Toxoplasma gondii*	*Mif^−^/^−^* mice (BALB/c and C57BL/6 systemic infection; virulent RH and avirulent ME49)	↓	MIF stimulates production of IL-1*β*, IL-12, TNF, NO, and IFN-*γ*	MIF prevents tissue pathology, including liver lesions, a finding associated with parasite burden control and reduction of mortality	[39]
	*Mif^−^/^−^* mice (C57BL/6; peroral infection ME49)		MIF controls parasite burden in ileum, while it increases TNF, IL-12, IFN*γ*, IL-23, and TGF-*β* and reduces IL-22 expression	MIF increases morbidity and mortality, increases MMP9 in ileum, contributes to its damage, and is involved in a sepsis-like response with liver impact	[40]
	*Mif^−/−^* (BALB/c peroral infection; ME49)		MIF improves maturation of DC and controls parasite burden in brain and livers	MIF prevents mortality	[41]

*Trypanosoma cruzi*	*Mif^−/−^* mice (BALB/c)	↓	MIF stimulates production of IL-1*β*, IL-12, and IL-18, Th1 polarization and specific IgG2a production	MIF prevents classical heart and striated muscle lesions, a finding associated with parasite burden control and prevention of mortality	[42]

*Plasmodium chabaudi *AS	*Mif^−/−^* mice (BALB/c); recombinant MIF	=	—	MIF inhibited erythropoiesis *in vitro* alone and synergizing with TNF and IFN*γ* *Mif^−/−^* had less severe anemia and increased survival	[43]

*Plasmodium chabaudi adami*	*Mif^−/−^* mice (BALB/c); Ab-neutralized MIF	↑	MIF promotes Th2 polarization (in its absence, cells react better to IL-12/anti-IL-4 with Th1 polarization)	—	[44]

*Plasmodium falciparum*	Human volunteers submitted to infection; correlation	=	—	MIF is associated with a number of circulating lymphocytes	[45]
	Infected children in endemic zone; correlation	↓	—	MIF concentrations in plasma and MIF produced by leukocytes *in vitro* inversely correlated with severity of malarial anemia	[46]
	Infected children in endemic zone; association between polymorphism of MIF promoter and pathology	=	—	MIF peripheral levels are associated with promoter polymorphisms and with susceptibility to severe malarial anemia	[47]
